# Automated and robust organ segmentation for 3D-based internal dose calculation

**DOI:** 10.1186/s13550-021-00796-5

**Published:** 2021-06-07

**Authors:** Mahmood Nazari, Luis David Jiménez-Franco, Michael Schroeder, Andreas Kluge, Marcus Bronzel, Sharok Kimiaei

**Affiliations:** 1 Technische Universität Dresden, Dresden, TU Germany; 2 ABX - CRO advanced pharmaceutical services, Dresden, Germany

**Keywords:** CT segmentation, Internal dosimetry, Automation, SPECT, 177Lu, Deep learning, Molecular radiotherapy (MRT)

## Abstract

**Purpose:**

In this work, we address image segmentation in the scope of dosimetry using deep learning and make three main contributions: (a) to extend and optimize the architecture of an existing convolutional neural network (CNN) in order to obtain a fast, robust and accurate computed tomography (CT)-based organ segmentation method for kidneys and livers; (b) to train the CNN with an inhomogeneous set of CT scans and validate the CNN for daily dosimetry; and (c) to evaluate dosimetry results obtained using automated organ segmentation in comparison with manual segmentation done by two independent experts.

**Methods:**

We adapted a performant deep learning approach using CT-images to delineate organ boundaries with sufficiently high accuracy and adequate processing time. The segmented organs were consequently used as binary masks for further convolution with a point spread function to retrieve the activity values from quantitatively reconstructed SPECT images for “volumetric”/3D dosimetry. The resulting activities were used to perform dosimetry calculations with the kidneys as source organs.

**Results:**

The computational expense of the algorithm was sufficient for clinical daily routine, required minimum pre-processing and performed with acceptable accuracy a Dice coefficient of $93\%$ for liver segmentation and of $94\%$ for kidney segmentation, respectively. In addition, kidney self-absorbed doses calculated using automated segmentation differed by $7\%$ from dosimetry performed by two medical physicists in 8 patients.

**Conclusion:**

The proposed approach may accelerate volumetric dosimetry of kidneys in molecular radiotherapy with 177Lu-labelled radiopharmaceuticals such as 177Lu-DOTATOC. However, even though a fully automated segmentation methodology based on CT images accelerates organ segmentation and performs with high accuracy, it does not remove the need for supervision and corrections by experts, mostly due to misalignments in the co-registration between SPECT and CT images.

*Trial registration* EudraCT, 2016-001897-13. Registered 26.04.2016, www.clinicaltrialsregister.eu/ctr-search/search?query=2016-001897-13.

## Introduction

The molecular radiotherapy (MRT) using tumour-targeting peptide pharmacophores, labelled with radioisotopes such as Lu-177 or Y-90, is increasingly used for treatment of targetable cancers such as neuroendocrine tumours (NETs) [1–3], or prostate cancer [4]. MRT has the advantage of offering more personalized cancer treatment as radiopeptides can be designed to the molecular characteristics of a tumour and deliver defined radiation doses to a specific targets. To optimize treatment, i.e. in order to safely administer MRT agents, various dosimetry methodologies have been developed to estimate and calculate the radiation doses delivered to various organs.

Medical Internal Radiation Dose (MIRD) is a commonly used method which determines the cumulative activity of organs of interest through various compartment models and the absorbed dose, estimated the s-values of phantom-based models [5]. The phantom-based dose estimators, however, lack [6] the specific patient and uptake geometry as the organs are standardized and a homogeneous activity distribution within each organ is assumed. To overcome these limitations, different patient-specific dosimetry methods have been adapted where the radiation dose is calculated on a voxel-by-voxel basis taking into consideration the individual organ shape and activity uptake.

Hybrid, also referred to as 2.5-dimensional (2.5D) dosimetry [7, 8], uses a series of planar (2D) images to generate time activity curves (TACs) for each organ of interest, which are subsequently calibrated by organ using the 3D effect factor from a single quantitative SPECT/CT scan. In 3D dosimetry, organ TAC is determined based on quantitatively reconstructed SPECT/CT series [9] using data from delineated organs obtained from multiple quantitative SPECT/CT time points. In a final step, the delivered dose is calculated by convolution of voxel-per-voxel cumulative activity of each organ with an energy deposition kernel (Voxel S) [10].

As described above, both 2.5D and 3D methodologies rely on delineated organs of interest. Therefore, the final estimated radiation dose deposited depends on the accuracy of the 3D organ delineation. One proposed way to obtain accurate organ boundaries is to perform segmentation on CT images. The resulting mask can further be applied to the corresponding SPECT data for activity extraction. Furthermore, to compensate the SPECT mask for the lower spatial resolution and partial volume effect, one adapted method has been to convolve the CT mask with a point spread function, prior to its application to the SPECT data.

Developing methods to segment organs from CT images remains a significant challenge [11]. Today, segmentation of anatomical images is still either done manually or or semi-automated [12] which is time-consuming, error-prone, operator-dependent and requires significant human expertise. The manual segmentation of a single organ is typically performed slice-by-slice using either an available free-hand contouring tool or an interactive segmentation method guiding the operator during the process [13].

Kidneys are typical organs of interest in MRT, and relatively easy to visually identify on CT scans, even without intravenous contrast [14]. Despite their visibility, kidney segmentation still remains a tedious procedure. Sharma et al. [15] estimated a duration of 30 min for an expert to segment one kidney.

Liver segmentation is an even more challenging task. Livers are large, inhomogeneous and vary considerably from one patient to another [16]. Standard CT-scans of livers suffer from blurry edges, due to partial volume effects and motion artifacts induced by breathing and heart beats, increasing the level of complexity during delineation. Manual or semi-automated segmentation of the liver require on average 60 to 120 min from a clinical CT scan with a slice thicknesses of 2 to 5 mm [17].

With the development of artificial intelligence (AI), various deep learning algorithms have been introduced that can fully or semi-automatically segment livers and kidneys with sufficiently high accuracy [18] but with considerably less human interaction and effort. The most potent and accurate of these algorithms operate in 3D, making them computationally expensive and therefore unsuitable for daily routine practice. Furthermore, it is still unclear to what extent delineation errors and discrepancies from manual segmentation are transferred to dose calculation and consequently impact the calculated absorbed radiation dose to organs.

In this paper we introduce a light-weight, yet robust and automated liver and kidney segmentation methodology based on the Mask-rcnn algorithm [19] that can be adapted to clinical routine practice, and does not require any dedicated hardware. We further analyse and discuss the impact of method-related error on final absorbed dose estimates to the kidneys, using Lu-177 DOTATOC treatment as an example.

## Materials and methods

In this section, we address datasets, the algorithm, data processing and training of the algorithm in details.

### Datasets

The CNN used in this work was trained and evaluated using databases as per the following: dataset 1, 2 and 3 were consisting of CT data obtained from various sources used individuality to train, evaluate and test the network. Dataset 4 consisted of SPECT/CT images intended for dosimetry evaluation.

#### Liver: dataset 1

Dataset 1 consisted of 170 abdominal CT scans from a liver CT-image repository, the LiTS dataset (Liver Tumour Segmentation Challenge) [20]. The image data was acquired with different acquisition protocols, CT scanners and highly variable resolution and image quality. The dataset was originally acquired by seven hospitals and research institutions and manually reviewed by three independent radiologists. The CT images had large variations in the in-plane resolution (0.55–1.0 mm) and slice spacing (0.45–6.0 mm). CT scans included a variety of pre- and post-therapy images [21].

#### Kidney: dataset 2

Dataset 2 consisted of multi-phase CT scans with in-plane resolution and slice thickness ranging from 0.437 to 1.04 mm and from 0.5 to 5.0 mm, respectively (KiTS19 Challenge database [22]). This dataset included 200 CT scans of patients with kidney tumours (87 female, 123 male). The dataset provided ground truth with different masks for tumour and healthy kidney tissue. During the training, we considered the tumour mask as part of the kidney. A detailed description of the ground truth segmentation strategy is described by Santini et. al. [23].

#### Kidney: dataset 3

Dataset 3 consisted of 12 patients with 12 contrast-enhanced CT scans and 48 low-dose abdominal CT scans. The image data was acquired with different acquisition protocols, CT scanners and highly variable resolution and image quality. The dataset was originally acquired by six hospitals in 5 different countries undergoing organ dosimetry in the context of a clinical trial (internal). The CT scans varied in in-plane resolution from 0.45 to 0.9 mm and slice spacing from 0.8 to 4.0 mm, respectively. The organ segmentation was done by a single medical physicist and confirmed by a certified radiologist. One major difference in comparison with dataset 2 was that dataset 3 did not include the renal pelvis, renal artery and renal vein as part of the kidney segmentation in contrast-enhanced CT and low-dose CT images.

#### SPECT/CT: dataset 4

Dataset 4 was used to evaluate the impact of automated segmentation on dosimetry outcome. The dataset consisted of images from 8 patients with neuroendocrine tumours treated with 1 cycle of 177Lu-DOTATOC (7.5 GBq/cycle) undergoing kidney dosimetry in the context of a clinical study (internal). Abdominal contrast-enhanced CT scans were used to determine the volume of both kidneys. Four (4) abdominal SPECT/CT scans with in-plane SPECT image size of $256 \times 256$ and Low-Dose CT (LDCT) scans with an in-plane size of $512 \times 512$ were acquired at 0.5 h, 6 h, 24 h, 72 h post injection (p.i.). Co-registration between the LDCT scans and the SPECT scans was verified by two separate medical imaging experts, and the images were further coregistered manually when needed.

### Segmentation

The CNN used in paper was a modified deep learning model inspired by Mask-rcnn [19] and operated in 2.5-dimensional (2.5D) mode. In 2.5D mode, a number of adjacent 2D axial slices, where the main slice is in the middle channel, are used as one input. The modified network algorithm operates in two steps. In the first step, the network proposes multiple Regions of Interests (RoIs) where the RoIs are given a score and are classified in a binary manner. In the second step, the positively classified RoIs, i.e. the RoIs that contain objects of interest are fine-tuned to better include the area where the object of interest is located. The objects of interest within the RoIs are multi-classified and binary-masked. The algorithm is further explained in the following section.

#### Algorithm design

**Fig. 1:**
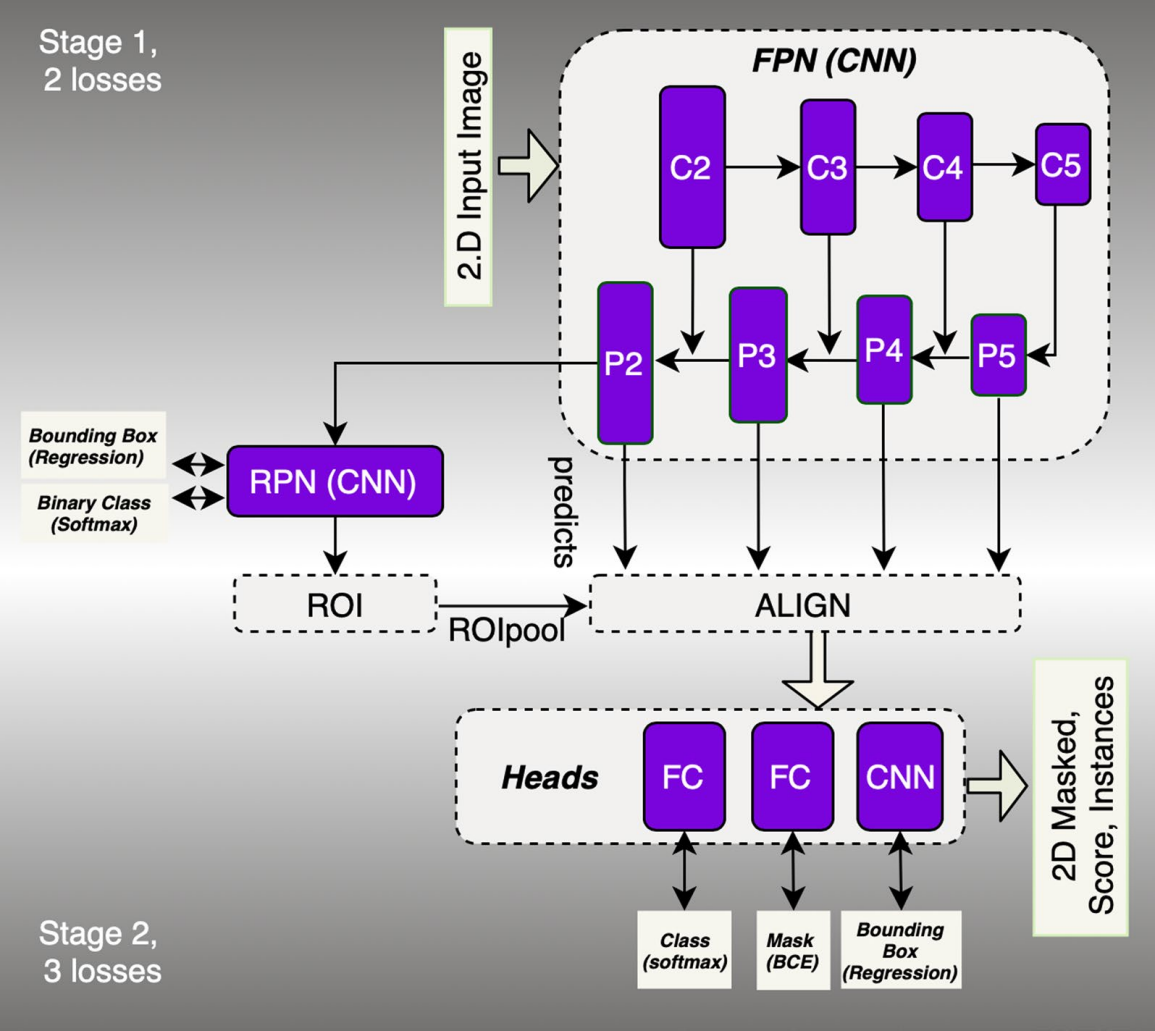
Mask-rcnn structure consists of two stages. The object of interest in the input image is artificially wrapped into boxes, binary-classified and fine-tuned. These boxes are then fed into the second stage of the network to be further fine-tuned to better fit the area where the object is located and multi-classified. Pixels inside the best box are then binary-classified to generate the mask. In this image, RPN stands for regional proposal network, FPN stands for feature pyramid network, RoI for region of interest and ALIGN is the RoI-Align mechanism. The head section is where 3 separate networks (two FCs, i.e. fully connected neural network and one CNN) generate the output. The rectangular boxes connected to the RPN box and Heads box indicate the type of loss functions. C and P represent the CNN layers used to construct the bottom-up and top-down architecture of the FPN respectively [26]

The Mask-rcnn structure is illustrated in Fig. 1 derived from Faster r-cnn [24]. The structure of Mask-rcnn consists of two stages: in the first stage, proposed regions where an object of interest might be located are boxed and binary-classified (i.e. if a box contains an object or not). In this stage, a process called *non-maximal suppression* binary-labels the boxes with the highest Intersection-over-Union (IoU) overlap with a ground-truth for further preparation of the training dataset. The training dataset, i.e. labelled boxes are then fed into a Regional Proposal Network (RPN) for training. The RPN is a method using CNN that scans features detected by backbone (the main structure of the network) referred to as FPN (Feature Proposal Network, the CNN layers where features are extracted). Thus, the RPN learns how to identify and box interesting objects, RoIs, in the input image. In the second step, localization of the RoIs is achieved by a mechanism called RoI-Align [19], aligning the extracted features with the input after the RoIPool [25]. RoIPool spatially normalizes the RoI features regardless of their size into a pre-defined space, e.g. $7 \times 7$.

In the inference mode, an algorithm trained through these steps can predict the bounding boxes, the segmented object as binary mask, the regression score as confidentiality score, and the classification. Further details of the algorithm are explained in “Appendix A.1”.

Quantitative evaluation of the segmentation process described was assessed by the Dice Score Coefficient (DSC). The proposed network was evaluated in two different modes. In the first mode, the images in the axial plane were fed as input to the algorithm and the accuracy was calculated as the global mean DSC for all corresponding slices. In the second mode, images in axial, sagittal and coronal planes were fed separately to perform segmentation prediction individually prior to a pixel-wise consensus procedure. Further details of the method are explained in “Appendix A.2”.

The major modifications in the Mask-rcnn structure were as follows: (I). we changed the input from 2D to 2.5D; (II); we increased the size of RoI-pooling from $7\times 7$ [27, 24] to $28\times 28$; (III); we decreased the binary mask size to $256\times 256$ from original ground truth size $512\times 512$. (II) was done to increase the precision of the error calculation in the first step of the network training at the expense of the memory consumption, and (III) was done to decrease memory consumption at the expense of lower precision for the error calculation in the second step of the network training. (IV) we did not use *P*1 and *C*1 for RPN, as we were aware that a kidney or a liver would not cover the whole field of view of a CT slice. All the modifications empirically showed $20\%$ decrease in memory consumption but 4 times reduction in speed for the specifications required in this task. The evaluation of the network without the modifications for liver segmentation resulted in an average $15\%$ lower test accuracy.

#### Pre and post processing

Despite the fact that different Hounsfield Unit (HU) values characterize different organs [28], these values often overlap for soft tissues, making the threshold-based discrimination of tissues or organs difficult [29]. To avoid the thresholding problem, the CT images were windowed by applying a threshold between $[-100,200]$ HU. This thresholding was the only pre-processing performed on the datasets.

In the mode where no consensus process is applied (refer to “Appendix A.2”) the algorithm failed to generate masks on LDCTs in an average of $2\%$ of the total number of single slices for each patient in validation and test datasets. By visual inspection of such slices, we observed that for liver, the delineation failed with higher probability where liver and heart were in the same plane. In kidney segmentation, the failure was not generalizable. In those cases, the missing masks were approximated by linear interpolation of the masks of the adjacent 2D-slices. Finally, in the inference mode where the test accuracy was calculated, the binary masks were resized using linear interpolation to the original size of the ground truth, i.e. from $256 \times 256$ to $512 \times 512$.

#### Algorithm training

The network was initially trained on a subset of images obtained from imageNet dataset (approx. 1 million non-medical images gathered for computer vision research and 1000 classes) [30] for 100 epochs (i.e. when the algorithm has trained on all the images/samples in the dataset) in order to train the backbone with the aim of learning the low semantic features. The trained algorithm (transfer learning [31, 32]) was further trained, evaluated and tested on each of the datasets 1–3 as described below. Dataset 4 was reserved for dose calculations and was not used during any training or testing. Furthermore, to enable the network for consensus mode, after the transfer learning process, the network was trained in all the 3 orthogonal planes simultaneously after the transfer learning process.

Training for the liver segmentation with dataset 1 was initially performed for 50 epochs by freezing (no training) the backbone and training the heads only with a learning rate ($\alpha$) of 0.001. This was done because we had only two classes in our task instead of 1000 used for imageNet training. It was followed by training the full network (backbone and heads) for 150 epochs with $\alpha = 0.0001$. Dataset 1 was used for the training, evaluation and test datasets with the ratio of 70/10/20 % for liver segmentation.

Training for kidneys was done in two stages. In the first stage, the network was trained for 50 epochs using dataset 2 by training the heads (freezing the backbone) with a learning rate $\alpha = 0.001$. The training was then continued with 100 epochs using the full network with $\alpha = 0.0001$. Up to this stage, $60\%$ of the dataset 2 was used for training, $20\%$ for validation and $20\%$ for test. In the second stage, using dataset 3, to fine-tune the network, i.e. with the purpose of teaching the network to exclude renal pelvis, renal artery and renal vein from segmentation, the heads were trained for 50 epochs on 10 CTs and evaluated on another 10 CTs each including 2 contrast-enhanced and 8 low-dose CTs belonging to 2 patients. After the full training, 40 CTs (8 patients) in dataset 3 were used for the calculation of the test accuracy.

Training time per epoch with a batch size of 2 was approximately 20 min using two Nvidia Titan XP GPUs. Furthermore, the network was trained, evaluated and tested 5 times (K-fold) [33], with random selection of the patients for training, validation and test subsets.

### Dosimetry

Dosimetric evaluations were performed using QDOSE ®software suite (ABX-CRO advanced pharmaceutical services, Germany). During the evaluations, Dose Volume Histograms (DVHs) of each kidney [34] were used as main measure to summarize the 3D absorbed dose distributions and to compare dose calculations between the algorithm and the calculations performed by the human experts.

The medical physicists, using dataset 4, applied the following procedure for safety dosimetry of the kidneys: the organ volumes were first determined by segmenting left and right kidneys, supervised using one of the manually or semi-automatic methods available in the software from the diagnostic CT scans. The delineated organs were then further used to calculate the masses of the kidneys assuming a density of 1.06 g/cc. The diagnostic CT scans were taken prior to the intravenous injection of 177Lu-DOTATOC. The activity concentrations in the kidneys at each time point post injection were then determined from the quantitative coregistered SPECT/CT images, where the kidneys were first delineated on the low-dose CT and then convolved with a point-spread function (Gaussian with sigma of 3*mm* ) for border extension. The same procedure was used for the evaluation of the automated segmentation with the network.

During volume determination of kidneys, the medical physicists segmented the renal parenchyma, representing the kidneys’ functional tissue, excluding the renal artery, renal vein and renal pelvis from the contrast-enhanced CT scans. For organ activity determination, the high activity concentration (renal) filtrate (i.e. urine containing the radiopharmaceutical/radioactive metabolites filtrated by the kidneys) was excluded when clearly discernible. The experts usually excluded the pelvis only at the first time point (0.5 h p.i.) when there was a high activity concentration in the filtrate.

Two independent experts performed the dosimetry calculations. Calculations for 5 patients were performed by expert 1 while the dose calculations for the other 3 patients (patient 5, 6 and 8) were performed by expert 2.

#### Dosimetry by expert 1

Expert 1 used the segmentation on the LDCT including border extension to obtain activity values from the corresponding SPECT images. The segmentation in the SPECT images was manually adapted (when needed) to avoid the inclusion of activity from other organs with high uptake (such as the spleen for some patients) or from tumour lesions (mostly hepatic lesions). This methodology was used on 5 patients as shown in the Tables 3 and 4. To be able to use this methodology, each SPECT and CT couple had to be coregistered to avoid mismatch between the images due to motion and breathing. The activity values obtained from the SPECT scans, 4 sets per patient, were fitted to a bi-exponential curve and integrated to calculate the time activity curve and the cumulated activity.

#### Dosimetry by expert 2

Expert 1 and expert 2 calculated the mass on the diagnostic CT images in the same manner. However, for the activity retrieval, expert 2 segmented the kidney VoIs directly on the SPECT by applying a threshold-based segmentation followed by manual correction when needed. Hence, expert 2 removed the necessity of co-registration between SPECT and CT for the 4 time points and provided a better consideration of the spill-out effect. The LDCTs were only used for verification purposes.

#### Dose estimation using AI segmentation

Kidneys were segmented by the network in the diagnostic CT to determine the masses for all 4 low-dose CT scans on dataset 4 using the network. The masks obtained from LDCTs were expanded by 3*mm* as explained previously and imported to QDOSE ®for dose calculations.

Dosimetric procedures to determine the cumulative activity values were identical as the methods used by expert 1 in “Dosimetry by expert 1” section, with the exception that the SPECT images were not adopted in order to avoid the inclusion of activity from other organs with high uptake.

## Results

**Fig. 2:**
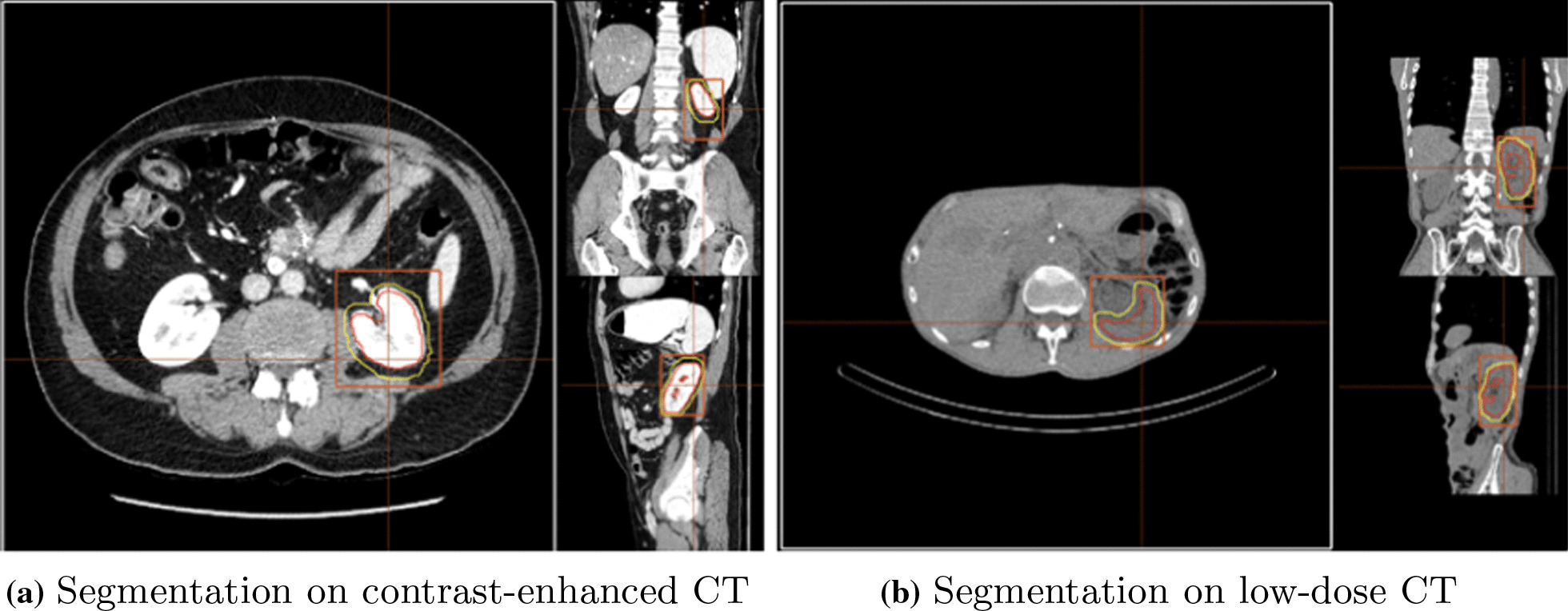
Segmented left kidney along axial, sagittal and coronal axis using the AI . The segmentation boundaries are highlighted with red contour on a contrast-enhanced CT on the left-hand side and on a low-dose CT on the right-hand side. The red rectangle corresponds to the bounding box used in kidney detection by the algorithm and the yellow contour is the 3 mm expanded region for activity retrieval from the SPECT images based on the CT-segmentation

Segmentation accuracy expressed as Dice score coefficient for segmented livers (using dataset 1) and kidneys (using dataset 2) is shown in Tables 1 and 2 in comparison with other top performing methods reported in the literature. An example of a segmented left kidney, using dataset 4, for both contrast-enhanced and low-dose CT images is shown in Fig. 2. The global Dice-coefficient accuracy obtained for the segmented livers was 93.40. The kidney accuracies for the first stage (dataset 2) were 94.10 and 94.60 for the second stage (dataset 3). The values reported are for the average of fivefold cross validations of the datasets. The accuracy achieved in the consensus mode shows an increase of up to $1.5\%$ in Dice score at the expense of independently running the network 3 times, thus triplication of the computational cost. In addition, the training without the transfer learning on ImageNet dataset provided on average $8\%$ and $6\%$ drops in accuracy on the test data for the liver and kidney, respectively, due to early over-fitting [35].

The average CPU time required to segment each of the 2.5D slices with the proposed algorithm on a 1.7 GHz Intel Core *i*7 was 2.5 s. The average time required to segment an entire liver as well as both kidneys using a standard gaming GPU (Nvidia GTx 1070) was less than 3 seconds.

**Table 1:** Liver segmentation accuracy

Method	Dice coefficient%	Tumour%	Method	Dataset
[36]	96.30	65.70	DL	1
[37]	95.90	50.01	DL	1
[38]	95.57	59.36	DL	1
[39]	94.30	72.00	DL	1
[40]	86.00	–	Non-DL	Internal
Our	93.40	–	DL	1

The accuracy reported is an average of 5 runs. The LiTS dataset, used for the calculations using the reported method, provides independent masks for the hepatic tumours. In our implementation, we combined the tumour masks and the liver masks to determine the total liver masks

DL, deep learning algorithm; non-DL, other methods

**Table 2:** Kidney segmentation accuracy comparison on KiTS19 dataset

Method	Dice coefficient%	Tumour %	Method	Dataset
[23]	98.00	73.00	DL	2
[41]	88.00	–	Non-DL	Internal
Our	94.10	–	DL	2

The reported accuracy is an average of 5 independent runs. The KiTS19 dataset provides independent masks for the kidney tumours. In our implementation, we combined the tumour masks and the kidney masks to determine the total kidney masks

DL, deep learning algorithm; non-DL, other methods

A comparison of kidney masses using automated segmentation, as determined versus those reported by experts (as ground truth) based on contrast-enhanced CT images from 8 patients (dataset 4), is shown in Table 3. The mean absorbed doses in the kidneys (mean dose to all voxels in the SPECT kidney masks) are shown in Table 4 for the same dataset and patients.

The differences in the mass calculations between AI and the experts for both kidneys in the patients 1 and 4 were higher than $12\%$. Thus, it was important to observe how these differences would impact the final calculation of the kidney doses.

The kidney doses are shown in Table 4. It can be seen that the AI method in patient 1 differed from the ground truth by underestimating the dose calculation by $2.5\%$. Similarly, there was an overestimation of $6.5\%$ for patient 4. In contrast, for patient 7, there was a mass underestimation of $2.5\%$ while the kidney dose was underestimated by $25\%$, which triggered additional analysis (“Discussion” section).

**Fig. 3:**
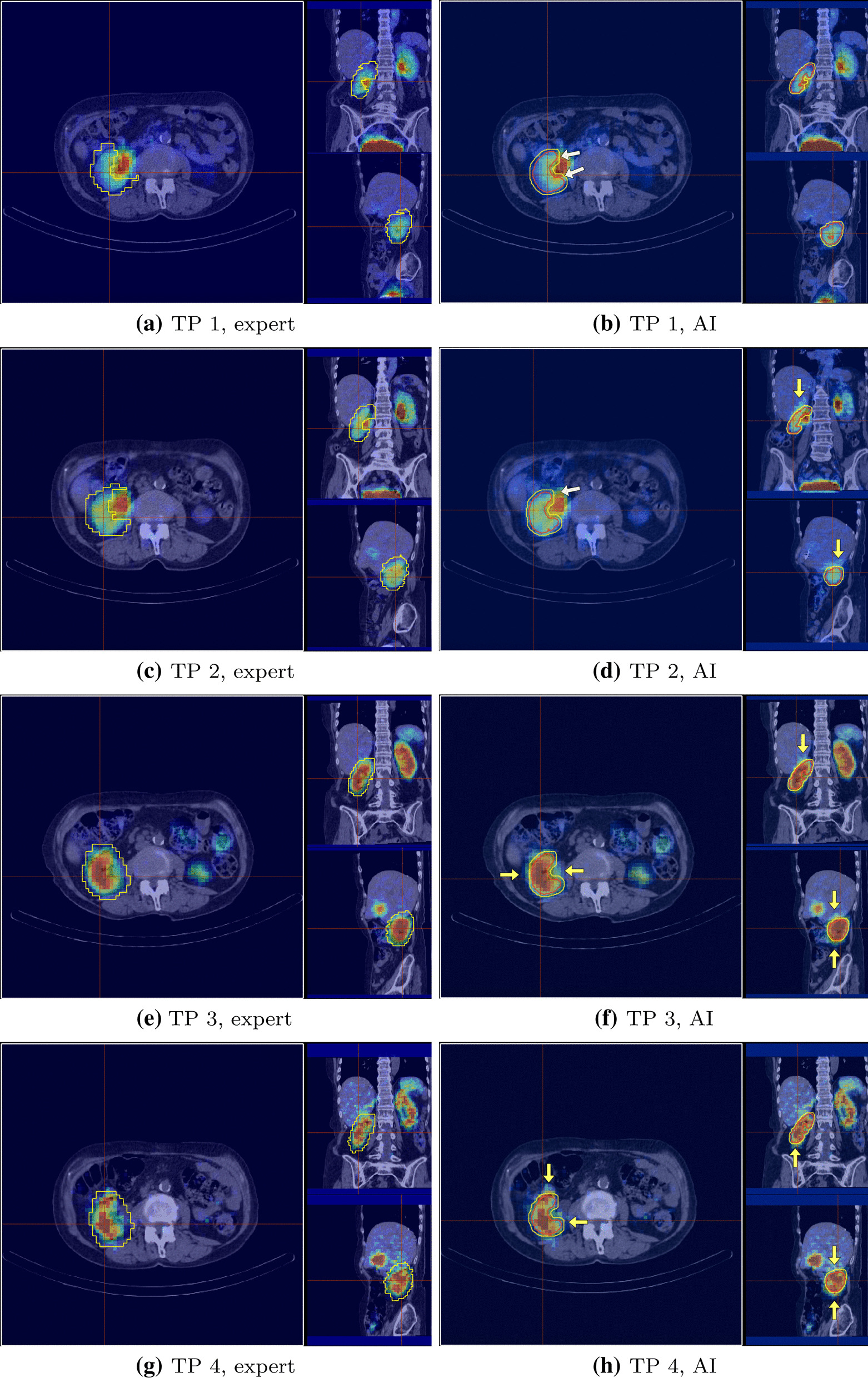
Comparison of the VoI segmentation of the right kidney of patient 7 based on the two different methodologies. Left: segmentation performed by expert 2. Right: segmentation when using the AI. The red contours illustrate segmentation on CT while the yellow contours show activity segmentation. Underestimated activity areas by the AI algorithm are pointed by a yellow arrow and overestimated activity areas by a white arrow

The SPECT/CT fused images for the 4 time points for patient 7 comparing the AI-based segmentation with the segmentation performed by expert 2 is shown in Fig. 3. The red contour in the Figure corresponds to the VoI segmentation using the CT image and the yellow corresponds to SPECT being used for segmentation.

**Table 3:** Calculated left and right kidney masses (g) based on AI (labelled with “AI”) and experts (labelled with “Ex”) segmentation on dataset 4

Mass/Patient	1	2	3	4	5	6	7	8	Avg.
L Kid Ex(g)	148	102	254	147	99	142	107	184	
R Kid Ex(g)	148	166	*	160	122	127	90	178	
L Kid AI(g)	169	93	243	125	95	138	102	188	
R Kid AI(g)	166	184	*	137	124	104	90	170	
L Kid Di(%)	14	(-) 9	(-) 4	(-)15	(-) 4	(-) 3	(-) 5	2	
R Kid Di(%)	12	11	*	(-)14	2	(-)18	0	(-) 4	
Mean Di(%)	13	10	4	14.5	3	10.5	2.5	3	7.5

The relative differences (labelled with “Di”), for left, right and average are shown in the last 3 rows. The segmentation is done on the contrast-enhanced CT taken prior to the radiopharmaceutical administration

(-) and * represent mass underestimation by the AI and lack of organ in the patient, respectively

**Table 4:** The calculated mean absorbed dose (Gy) deposited to the left and right kidneys resulted from application of the AI segmentation (labelled with “AI”) and the dose calculations performed by the experts (labelled with “Ex”)

Dose/Patient	1	2	3	4	5	6	7	8	Avg.
L Kid Ex(Gy)	1.79	1.72	1.89	1.77	2.83	3.49	2.19	2.00	
R Kid Ex(Gy)	1.61	1.50	*	1.57	2.52	3.39	2.66	2.01	
L Kid AI(Gy)	1.77	1.56	1.85	1.82	2.82	3.34	1.74	2.16	
R Kid AI(Gy)	1.55	1.42	*	1.73	2.54	3.56	1.87	2.08	
L Kid Di(%)	(-) 1	(-) 9	(-) 2	3	0	(-) 4	(-)20	8	
R Kid Di(%)	(-) 4	(-) 5	*	10	1	5	(-)30	3	
Mean. Di(%)	2.5	7	2	6.5	0.5	4.5	25	5.5	6.7

The relative differences (labelled with “Di”), for left, right and average are shown in the last 3 rows. (-) and * represent mass underestimation by the AI and lack of organ in the patient, respectively

**Fig. 4:**
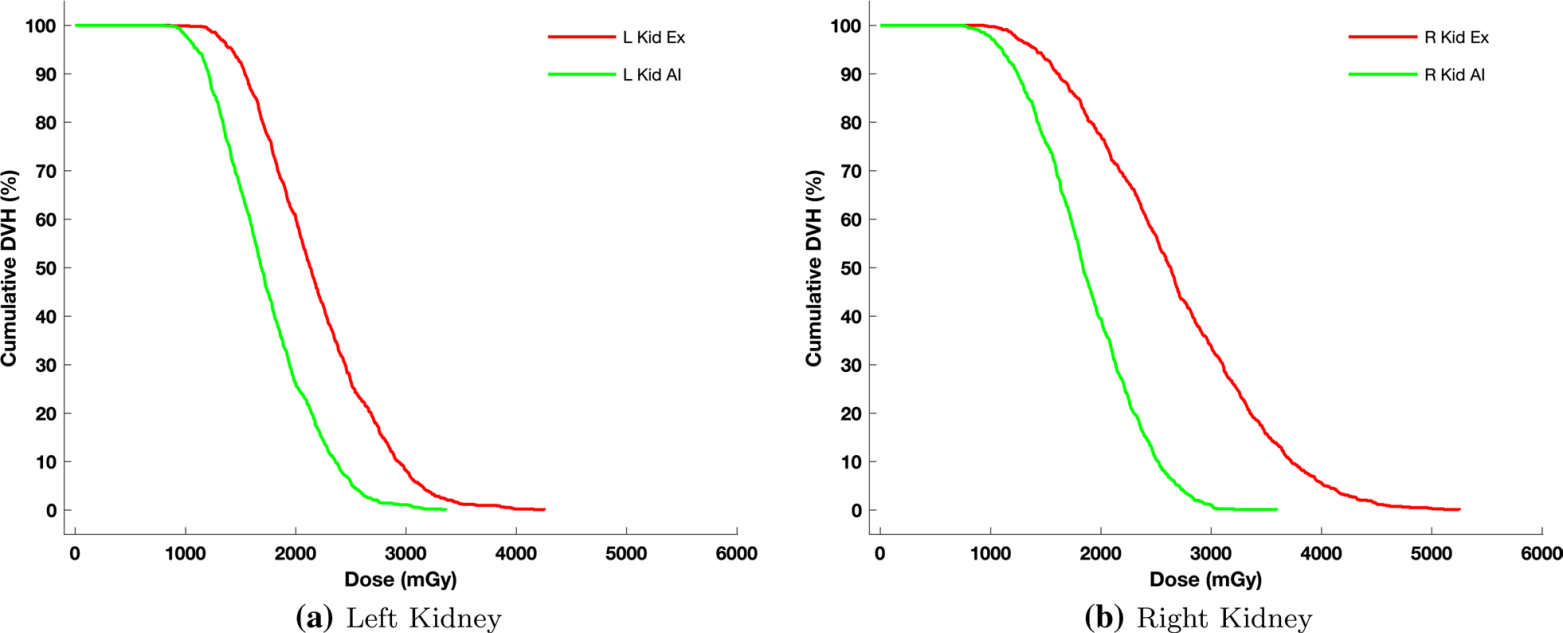
Dose volume histograms of left and right kidneys for patient 7 with the highest error margin. Red lines represent the dose calculations based on expert segmentation and the green lines represent the corresponding dose based on the AI segmentation

## Discussion

Using AI-based segmentation for organ delineation in volumetric dosimetry can be a cost-effective and powerful tool for personalized dosimetry, accelerating the dosimetry process from hours to minutes. The accuracy of the two-stage AI algorithm used in this paper is comparable with state-of-the-art algorithms as it was originally designed to perform instance segmentation in real time. Additionally, it can be run on a single-CPU laptop, with reasonable performance, as it is computationally cheaper. Another benefit of the two-stage structure presented here is the elimination of the spatial normalization of CT data, which is the normal practice for training deep learning algorithms, making the presented method more robust and scanner-independent. Training using 5 loss functions (“Appendix A.3”) makes the network slower during the training but faster during the inference mode which is beneficial during for daily practice. By simultaneously training the algorithm in the 3 orthogonal planes, the run time is threefold, but it allows the network to run in consensus mode which increases the robustness of the algorithm. In comparison, fully 3D structured CNNs such as [42, 43] can better leverage the spatial information along the third dimension and result in higher accuracy, but they introduce higher computational expense. The computational expenses however might not be an issue in the near future.

The kidney doses when using DL-organ segmentation AI differ from the dose calculations performed by the expert by $< 3\%$ for $\approx 40\%$ of the patients, and by $\le 7\%$ for $\approx 90\%$ of the patients. However, a deviation of $25\%$ for patient 7 was observed between two methods that required further analysis.

Further investigation of the deviating case (patient 7) revealed that the retrieved activities at time point 2 (Fig. 3d) time point 3 (Fig. 3f) and time point 4 (Fig. 3g) were considerably different. The discrepancy was due to the differences in the segmentation procedure between expert 2 and the AI-based method for that specific patient; while expert 2 considered a larger spill out effect than the estimated 3mm, the AI-based method strictly used 3mm as spill-out boundary on all CT-derived contours.

Furthermore, by investigating the Dose Volume Histograms (DVH) shown in Fig. 4, DVH, it can be seen that the DVH-70 and DVH-30, for the right kidney, were 1.6 and 2.1Gy, respectively, when using AI while the corresponding values when experts performed the segmentation were 2.2 and 3.1Gy. In addition, the decent of the slope for the AI method is steeper. For the left kidney, the decent of the slope is more similar between the two methods (Fig. 4a). The corresponding DVH-70 and DVH-30 for the left kidney were 1.4 and 2.0 Gy for the AI method while for the expert, these values were 1.8 and 2.5 Gy. The differences between the expert and the AI could be explained by inter-variability between the experts and misalignment between SPECT and LDCT due to motion.

To further investigate the misalignment, a spill out margin of 6mm was applied when using the AI-based segmentation method. The results obtained were a mean dose of 2.13 Gy for the left kidney and 2.37 Gy for the right kidney, respectively, i.e. $2.73\%$ and $10.90\%$ (average $6.8\%$) underestimation for the left- and right kidneys, respectively, which is more consistent with the remaining of results reported in table 4.

Although the main limitation of this study is the small number of patients in dataset 4, the obtained results are promising and indicate that automated segmentation may be successfully used for kidney delineation in daily dosimetry practice for patients undergoing MRT procedures with potentially nephrotox 177Lu-labelled radio-peptide therapeutic. Precise co-registration of SPECT images with their corresponding LDCT images is required for accurate activity extraction to minimize the impact of motion artifacts.

## Conclusion

We adapted a performant deep learning approach, initially designed for natural image segmentation, to be used on contrast-enhanced and low-dose CT images to calculate organ boundaries with acceptable accuracy and processing time. The collaboration of 5 loss functions executed in a two-stage network accelerated the processing time required and eliminated the need of pre-processing CT scans. The 2.5D algorithm implemented provides a fast and memory-efficient segmentation method and the additional voxel-based consensus algorithm presented made the model more robust and less error prone providing comparable results to more computationally expensive state-of-the-art 3D DL algorithms.

Our evaluation shows that the proposed approach is a promising method that may accelerate volumetric dosimetry of kidneys in patients undergoing MRT with renally excreted radio-peptides labelled with 177Lutetium. However, even though a fully automated segmentation methodology based on the CT-images only accelerates the organ segmentation burden, it does not fully remove the need for the supervised corrections as explained. A suggestion to overcome this limitation is to use the functional information (i.e. corresponding SPECT data) as complementary information during the training of the algorithm. This additional input could be incorporated to the AI algorithm as an extra channel of our 2.5D input image.

## Data Availability

Datasets 1 and 2 analysed during the current study are available in the: Dataset 1: codalab, https://competitions.codalab.org/competitions/17094. Dataset 2: grand-challenge, https://kits19.grand-challenge.org Datasets 3 and 4 analysed during the current study are not publicly available due to confidentiality of the study from which the data was extracted.

## A Algorithm

In the following sections, the algorithm is described in more details.

### A.1 Algorithm design

The Mask-rcnn derives from Faster r-cnn [24] and detects different objects in an image or a video, and also discriminates different instances of the same object (instant segmentation). The main differences between Faster-rccn and Mask-rcnn are that the latter generates a segmentation mask and localizes the mask more precisely on the input image. The generation of the mask is done by an extra branch, i.e. a connected convolutional neural network (CNN) which predicts the mask. Better localization than Faster r-cnn is achieved by a mechanism called RoI-Align [19] which properly aligns the extracted features with the input after the RoIPool [25]. Thus, using the image as an input, the algorithm delivers the segmentation, bounding boxes (the coordination of the RoI in the input image), regression score as confidentiality score, type of prediction (classes) and masks.

The structure of Mask-rcnn consists of two stages, shown in the Fig. 1. In the first stage, proposed regions where an object of interest might be located are artificially boxed, binary classified (if a box contains an object of not) and fed into the second stage. In the first stage these boxes are generated by drawing random rectangular shapes referred to as bonding box in the input image. The boxes have different aspect ratios and sizes based on the concept of Anchor (predefined bounding boxes of a certain height and width) [24] and are referenced to a point in the image e.g. middle coordination. The boxes are then filtered through a mechanism called non-maximal suppression. Non-maximal suppression binary-labels the boxes with the highest Intersection-over-Union (IoU) overlap with a ground-truth, i.e. boxes with IoU overlap higher than 0.7 and lesser than 0.3 with any ground-truth are binary labelled as 1 and 0, respectively. The rest of the bounding boxes are discarded. Boxes are then delivered into a Regional Proposal Network (RPN) for training. The RPN is a mechanism implemented using CNNs that scans feature maps (CNN filters) in the backbone (the main structure of the network) referred to as Feature Pyramid Network (FPN) [44, 45]. RPN scans the feature maps based on the size of the boxes, i.e. for bigger size boxes representing the bigger objects in the image the RPN referees the higher level of the CNN structure with higher semantic features (i.e. meaningful, the higher CNN layers have higher abstract features) of the feature maps, e.g. *P*5, while for smaller size boxes, the RPN referees the lower semantic features in the lower layer e.g. *P*2. These two loss functions are labelled as bonding box and binary class in Fig. 1 for RPN.

Feature maps scanned by RPN are generated by FPN. FPN is the backbone of the Mask-rcnn structure design, in our model designed with the ResNet50 model [46]. FPN is a CNN structure generating semantic-rich feature maps with high resolution objects and spatial information. C boxes in the Fig. 1 represent the bottom-up CNN layers for Resnet, i.e. down-sampling (max-pooling and stride of 2) the input while P boxes represent top-down (up-sampling) CNN layers [26]. Outer layers in the FPN such as *P*2 structure detect low semantic features with high resolution such as edges of a kidney while the deeper layers, e.g. *P*5, detect higher semantic features with low resolution such as the whole kidney. The top-down pathway, $P2{-}P5$ are enhanced with feature-lateral connections from bottom-up pathway, $C2{-}C5$ in Fig. 1. The lateral connections ($1 \times 1$ CNN layer) between top-down and bottom-up are used for better location of the features. We did not use *P*1 and *C*1 for RPN in our implementation as with experimentation we found that it slows down the inference mode with no increases in the performance. Since the boxes proposed by RPN have different scales, they are then scaled equally by a mechanism called RoI pooling which uses max pooling. Max pooling converts the features inside any valid RoI box into a fixed and smaller feature map, a fixed spatial extent embedded with float values [24], in our model $28 \times 28$ dimension, i.e. regardless of the RoI box size all RoIs are translated into the $28 \times 28$ box size. For our implementation this is 16 times larger than the value proposed in the original paper which resulted in better accuracy but highest computational cost based on our evaluation. The fixed scaled feature maps generated by RoI pooling are then better aligned by an alignment mechanism (ALIGN in Fig.1) which is used to re-align the position of a pixel regarding the original image. This is done to overcome the problem of shifting pixel positions due to frequent down- and up-scaling of the image executed in the backbone.

In the second stage, classified boxes acquired from the first stage are then refined, multi-classified (in our implementation binary-classified), binary-masked and are given a confidentiality score. That is, the shape of each proposed box from the first stage is fine-tuned (re-shaped) in order to better cover the RoI, multi-classify by instance segmentation of different classes and provide with a value $\in [0$ $100]\%$ to represent how “confident” the network is about the classification. We set the confidential score to $90\%$ for the final object detection during training and testing i.e. any kidney or liver with a lower score is discarded. These 3 different tasks are done with 3 separate Artificial Neural Networks (ANNs) known as heads; a CNN structure for mask classification and two different FCNN refereed as FC (Fully Connected) for regression and multi-classification, shown in Fig. 1 in the “Heads” section. Finally, the dimension of the binary mask generated by the heads was set to $256 \times 256$ to decrease the computation expenses. These masks then were linearly interpolated to $512 \times 512$ for the test dataset in the inference mode.

### A.2 Accuracy calculation

Quantitative evaluation of our segmentation algorithm was assessed by the Dice Score Coefficient (DSC) shown in Eq. 1. The segmentation predicted by the network ($S_{Pre}$) was pixel-wise compared with the ground truth segmentation ($S_{GT}$ ).

1 $$\begin{aligned} \hbox {Dice}(S_{\hbox {Pre}}, S_{\hbox {GT}})= \frac{2\cdot ( S_{\hbox {Pre}}\bigcap S_{\hbox {GT}})}{\left| S_ {\hbox {Pre}}+ S_{\hbox {GT}}\right| } \end{aligned}$$

Our network operates in two different modes. In the first mode, the images in the axial plane can be fed as input to the algorithm and the accuracy is calculated as the global mean DSC between all the slices. In the second mode, images in axial, sagittal and coronal planes are fed separately to perform segmentation prediction individually and then a pixel-wise (voxel) consensus procedure Eq. 2 takes place between all 3 predictions to make a 3D mask. Thus, if at least two of the predictions are positive for a given voxel (1), then the voxel is set to be positive; otherwise the voxel is set to be negative (0).

In Eq. 2, x, y, z represent a predicted voxel by feeding the network along the axial, sagittal and coronal planes, respectively. $p_{xyz}$ is the final result after the consensus procedure for that specific pixel.

2 $$\begin{aligned} p_{xyz},x,y,z \in \{0,1\}; p_{xyz} = xy \vee xz \vee yz \end{aligned}$$

### A.3 Loss

The network includes 5 loss functions which are jointly trained. Two loss functions are used in the first stage. One of them is to be trained with for fitting the rectangular object proposed boxes around the RoI $L_{box_1}$ as a regression loss function and the second one to binary-classify $L_{cls_1}$ the boxes (e.g. kidney or non-kidney as a binary classification loss).

In the second stage of the network, there are 3 loss functions. The first one is a categorical cross-entropy for multi-classification ($L_{cls_2}$), the second one is a regression loss ($L_{box_2}$) and the third one is a binary cross-entropy loss ($L_{mask}$) to calculate the binary mask of the target organ. The network’s main loss is a multi-task loss calculated as $L = L_{cls} + L_{box} + L_{mask}$.

$L_{mask}$ is defined as the average Binary Cross-Entropy (BCE) loss and generates masks for every class without competition between classes on the boxes received from the first stage. The bounding loss is $L_{box} = L_{box_1} + L_{box_2},$ and the classification loss is $L_{cls}= L_{cls_1}+ L_{cls_2}$.

The classifications loss values $L_{cls_1}$ and $L_{cls_2}$ are dependent on the confidence score of the true class, hence the classification loss functions reflect how confident the model is when predicting the class labels. The bounding box loss values $L_{box_1}$ and $L_{box_2}$ reflect the distance between the true box parameters (height and width) to the predicted ones as a regression loss function and the mask loss function $L_{mask}$, is similar to the classification loss function $L_{cls_1}$. It is the binary cross-entropy which performs the voxel-wise classification of those voxels inside the predicted (learned) box by $L_{box_2}$.

## Abbreviations

AI: Artificial intelligence
CNN: Convolutional neural network
CT: Computed tomography
DSC: Dice score coefficient
DVH: Dose volume histogram
FPN: Feature proposal network
HU: Hounsfield unit
IoU: Intersection-over-union
KiTS: Kidney tumour segmentation challenge
LDCT: Low-dose computed tomography
LiTS: Liver tumour segmentation challenge
MRT: Molecular radiotherapy
NET: Neuroendocrine tumours
RoI: Region of interest
RPN: Regional proposal network
SPECT: Single-photon emission computed tomography
TAC: Time activity curve
